# Current trends in basic research on Parkinson’s disease: from mitochondria, lysosome to α-synuclein

**DOI:** 10.1007/s00702-024-02774-2

**Published:** 2024-04-13

**Authors:** Hideaki Matsui, Ryosuke Takahashi

**Affiliations:** 1 Department of Neuroscience of Disease, Brain Research Institute, Niigata University, 1-757, Asahimachidori, Chuoku, Niigata 951-8585 Japan; 2 Department of Neurology, Kyoto University Graduate School of Medicine, Kyoto University, 54, Shogoin Kawahara-cho, Sakyoku, Kyoto, 606-8507 Japan

**Keywords:** Parkinson’s disease, Mitochondria, Lysosome, Vesicular transport, α-synuclein

## Abstract

Parkinson’s disease (PD) is a neurodegenerative disorder characterized by progressive degeneration of dopaminergic neurons in the substantia nigra and other brain regions. A key pathological feature of PD is the abnormal accumulation of α-synuclein protein within affected neurons, manifesting as Lewy bodies and Lewy neurites. Despite extensive research efforts spanning several decades, the underlying mechanisms of PD and disease-modifying therapies remain elusive. This review provides an overview of current trends in basic research on PD. Initially, it discusses the involvement of mitochondrial dysfunction in the pathogenesis of PD, followed by insights into the role of lysosomal dysfunction and disruptions in the vesicular transport system. Additionally, it delves into the pathological and physiological roles of α-synuclein, a crucial protein associated with PD pathophysiology. Overall, the purpose of this review is to comprehend the current state of elucidating the intricate mechanisms underlying PD and to outline future directions in understanding this disease.

## Introduction

Parkinson’s disease (PD) is a neurodegenerative disorder characterized by the gradual neuronal loss of the substantia nigra dopaminergic neurons and other brain areas, leading to movement and non-movement disorders (Kalia and Lang 2015). A prominent hallmark of PD pathology is the abnormal aggregation of α-synuclein protein, which forms aggregates known as Lewy bodies and Lewy neurites (Spillantini et al. 1997). Research to understand the pathogenesis of PD has been ongoing for a long time, and it is a relief to patients that several treatments are available. On the other hand, there is still no drug that is certain to slow the progression of the disease, and further progress in understanding the pathogenesis of PD is desirable. Here, we focus on basic researches on PD in the approximately 12-month period through May 2023. Initially, we summarize the impairment of mitochondrial-lysosomal function in the pathophysiology of PD. Subsequently, we present the latest findings on the involvement of α-synuclein in the interaction with these dysfunctions.

## Mitochondrial factors in PD pathogenesis

PD is a complex neurodegenerative disorder that results from the interplay of genetic and environmental factors. Several genes including *SNCA*, *LRRK2*, *GBA*, *PARK2*, *PARK7*, *PINK1*, and *VPS35* have been identified as causative or risk factors for PD, either in Mendelian or non-Mendelian forms (Funayama et al. 2023). Upon examining the genes associated with PD reported to date, it becomes evident that a central focus lies on those related to mitochondria, the autophagy-lysosome system, and vesicular transport processes involving both. This section begins by exploring recent research on genes strongly associated with mitochondria.

*PINK1* and *PRKN* are two genes that have been implicated in autosomal recessive PD and these genes have shed light on the process of selective autophagy-lysosomal degradation of damaged mitochondria, known as mitophagy, as a significant factor in the development of the disease (Kitada et al. 1998; Valente et al. 2004; Narendra et al. 2008). Soutar et al. conducted a mitophagy screening assay to investigate the functional significance of genetic risk genes identified in Genome-wide association study (GWAS). They discovered two new regulators, KAT8 and KANSL1 (KAT8 regulatory NSL complex subunit 1), that influence the initiation of PINK1-dependent mitophagy, a process associated with PD (Soutar et al. 2022). The findings suggest that PINK1-mitophagy pathway can be involved in idiopathic PD, and KANSL1 plays a critical role in the disease pathogenesis. Another group has also extracted *KANSL1* gene as a result of GWAS focusing on short tandem repeats (STRs) in PD (Bustos et al. 2023). STRs are small repetitive units involving a repetitive unit of 1–6 base pairs that vary among individuals (Chintalaphani et al. 2021). The authors analyzed data from 39,087 individuals, including PD cases and controls of European ancestry, and identified 34 genome-wide significant STR loci associated with PD. The strongest signal was found in the *KANSL1* gene. Gene expression analysis revealed that some STRs influenced the expression of multiple genes, including known PD-related genes.

Loss-of-function mutations in the *PARK7* gene, which encodes DJ-1, respectively, is also associated with autosomal recessive forms of PD (Bonifati et al. 2003). While the roles of PINK1 and parkin in mediating mitophagy are well established, the mechanisms by which DJ-1 loss leads to PD are not fully understood. Imberechts et al. investigated PINK1/parkin-mediated mitophagy in human fibroblasts and iPSC-derived neurons with homozygous PARK7 mutations (Imberechts et al. 2022). The study found that DJ-1 is crucial for PINK1/parkin-mediated mitophagy. Loss of DJ-1 did not affect the activation of PINK1 or parkin after mitochondrial depolarization but instead disrupted mitophagy downstream by inhibiting the recruitment of the selective autophagy receptor optineurin to depolarized mitochondria. DJ-1 translocated to depolarized mitochondria in close proximity to optineurin, and this translocation depended on PINK1 and parkin. Overexpression of DJ-1 could not rescue the mitophagy defect in cells lacking PINK1 or parkin, suggesting DJ-1 functions downstream of PINK1 and parkin in the same pathway specifically in mediating mitophagy. Disruption of PINK1/parkin/DJ-1-mediated mitophagy may be a common pathogenic mechanism in autosomal recessive PD.

Whole exome sequencing (WES) is a genomic technique that focuses on sequencing the protein-coding regions of the genome. It allows for the identification of genetic variants within these regions, including disease-causing variants. Li et al. performed WES on a cohort of 1917 patients with early-onset or familial PD and a cohort of 1962 patients with sporadic late-onset PD (Li et al. 2022). They identified a significant association between *PPARGC1A* rare missense variants and susceptibility to early-onset and familial PD. PPARGC1A is considered a master regulator of mitochondrial biogenesis, but the functional relationship between this gene and PD awaits further investigation in future research (Piccinin et al. 2021).

A recent study also focused on pacemaking substantia nigra dopaminergic neurons and used various techniques to investigate their mechanisms (Zampese et al. 2022). They found that calcium entry through L type voltage-gated calcium channels, triggered by neuronal spikes, led to calcium release from the endoplasmic reticulum. This calcium release stimulated mitochondrial oxidative phosphorylation through two separate Ca2+-dependent mechanisms: the mitochondrial uniporter and the malate-aspartate shuttle. Disrupting either of these mechanisms impaired the ability of dopaminergic neurons to sustain spike activity. While this feedforward control helps dopaminergic neurons meet their energy demands during sustained spiking, it also contributes to increased oxidative stress, which may be associated with aging and disease-related decline in these neurons.

The discussion related to mitochondrial dysfunction in PD mostly revolves around pathways associated with the degradation system of impaired mitochondria. The discussion on α-synuclein will be addressed later, but it is noteworthy that in familial PD caused by genes related to mitochondria or in animal models induced by compounds with mitochondrial toxicity, there is not necessarily a significant accumulation of α-synuclein in the brain. While the mechanism behind this phenomenon requires explanation, it is also plausible to consider that PD may involve a mixture of several distinct diseases (Ahlskog et al. 2009), though conclusive statements cannot be made at present.

## Lysosomal factors in PD pathogenesis

Next, our focus will shift to the autophagy-lysosome system. *GBA* gene, which encodes the enzyme β-glucocerebrosidase (GCase) and is linked to Gaucher’s disease and PD (Sidransky et al. 2009). GCase is a lysosomal enzyme, and two recent papers have presented the relationship between GCase and mitochondria. Baden et al. discovered that GCase can enter the mitochondria from the cytosol by recognizing internal mitochondrial targeting signals (Baden et al. 2023). Within the mitochondria, GCase plays a role in maintaining the integrity and function of mitochondrial complex I, which is involved in energy metabolism. GCase also interacts with mitochondrial quality control proteins HSP60 and LONP1. Disease-associated mutations in *GBA* disrupt complex I stability and function, and enhance the interaction with the mitochondrial quality control machinery. By using a multi-part enrichment proteomics and post-translational modification (PTM) approach, Bogetofte et al. 2023 identify numerous dysregulated proteins and PTMs in dopamine neurons derived from iPSCs of PD patients with heterozygous GBA-N370S mutation (Bogetofte et al. 2023). They found disturbances in glycosylation and the autophagy-lysosomal pathway, accompanied by perturbations in mammalian target of rapamycin (mTOR) activation in GBA-PD neurons. Dysregulation of proteins encoded by PD-associated genes is also observed. Integrated pathway analysis reveals impaired neuritogenesis, with tau identified as a key pathway mediator. Functional assays confirm deficits in neurite outgrowth and impaired mitochondrial movement in GBA-PD neurons. Pharmacological rescue of glucocerebrosidase activity using NCGC758 improves the neurite outgrowth deficit.

ATP13A2 is a lysosomal ATPase associated with a familial PD and several reports have been published on its ability as a transporter (Ramirez et al. 2006), and a recent report claims that ATP13A2 is an H+,K+-ATPase (Fujii et al. 2023). The ATPase activity and K+-transport activity of ATP13A2 were found to be inhibited by certain inhibitors of sarco/endoplasmic reticulum Ca2+-ATPase and gastric H+,K+-ATPase. Remarkably, these H+,K+-ATPase inhibitors induced lysosomal alkalinization and accumulation of α-synuclein. Additionally, the study revealed that mutations in ATP13A2 associated with PD lead to abnormal expression and function. These findings suggest that the H+/K+-transporting function of ATP13A2 plays a role in maintaining lysosomal acidity and facilitating α-synuclein degradation within lysosomes. TMEM175 is a lysosomal channel and *TMEM175* mutations have been identified by several GWASs as a genetic risk factor for PD (Chang et al. 2017; Blauwendraat et al. 2019; Krohn et al. 2020). Hu et al. demonstrate that TMEM175 acts as a proton-activated, proton-selective channel on the lysosomal membrane, mediating the H + leak necessary to maintain the optimal acidic pH range in lysosomes (Hu et al. 2022). Activation of TMEM175 prevents excessive acidification of lysosomes. Both endogenous and synthetic agonists can activate TMEM175 and trigger the release of protons from lysosomes. Deficiency in TMEM175 leads to lysosomal over-acidification, impaired proteolytic activity, and increased aggregation of α-synuclein.

Senkevich et al. 2023 identified an association between the *GALC* gene, which encodes the lysosomal enzyme galactosylceramidase, and PD (Senkevich et al. 2023). The research demonstrated that a specific variant in the *GALC* locus is associated with increased galactosylceramidase activity and PD.

The functional relationship between *GALC* and the pathogenesis of PD awaits further investigation.

As one idea for how lysosomal dysfunction induces PD, there is a vicious cycle hypothesis suggesting that when lysosomal function is impaired, α-synuclein accumulates, and further impairment of lysosomal function occurs as α-synuclein accumulates (Bellomo et al. 2020). However, it is crucial to note that in GBA-deficient medaka, degeneration of dopaminergic neurons is observed regardless of the presence of α-synuclein. Additionally, in zebrafish, which inherently lack α-synuclein, degeneration of dopaminergic neurons is observed with GBA deficiency or ATP13A2 deficiency, underscoring the need for careful consideration (Uemura et al. 2015; Nyuzuki et al. 2020; Matsui et al. 2021). Given these experimental results, it may be more natural to assume that lysosomal dysfunction leads to an accumulation of impaired mitochondrial components or itself, and it can be inferred that a vicious loop might easily form between lysosomal dysfunction and mitochondrial impairment (Stepien et al. 2020).

## Vesicular transport factors in PD pathogenesis

Vesicular transport is a cellular process that involves the movement of substances within a cell through small, membrane-bound structures called vesicles. These vesicles, encapsulated by membranes, serve as carriers for transporting various molecules between different cellular compartments or between the cell and its external environment (Smolders et al. 2020). *LRRK2*, *VPS35*, *VPS13C*, *DNAJC6* and *SYNJ1* are genes strongly associated with vesicular transport among the molecular components related to PD.

*LRRK2* is a gene that encodes a protein kinase enzyme. It is associated with PD, as mutations in the *LRRK2* gene are a known genetic cause of inherited forms of the disease and genetic risk of sporadic PD (Paisan-Ruiz et al. 2004; Zimprich et al. 2004; Satake et al. 2009; Simón-Sánchez et al. 2009). Three recent studies provide insights into the complex functions and dysregulation of LRRK2 in PD, particularly in relation to intracellular membrane dynamics. In the first study, the researchers directed LRRK2 to lysosomes and early endosomes, resulting in autophosphorylation of LRRK2 itself and phosphorylation of its direct substrates Rab10 and Rab12 (Kluss et al. 2022). The phosphorylation of Rab10 was primarily observed in perinuclear lysosomes, while Rab12 phosphorylation occurred in both peripheral and perinuclear LRRK2-positive lysosomes. This suggests that lysosomal localization of LRRK2 plays an important role in regulating LRRK2-dependent Rab phosphorylation. The researchers found that anterograde transport of lysosomes to the cell periphery hindered the recruitment and phosphorylation of Rab10 by LRRK2. The absence of phosphorylated Rab10 from the lysosomal membrane prevented the formation of lysosomal tubulation and sorting processes. On the other hand, clustering of lysosomes in the perinuclear area increased recruitment and phosphorylation of Rab10 by LRRK2. These findings suggest that LRRK2 can be activated at various cellular membranes, including lysosomes, and that lysosomal positioning further regulates specific Rab substrates, Second study demonstrated that hyperphosphorylated Rabs by LRRK2 disrupted the transport of autophagosomes along axons by disturbing the coordinated regulation of cytoplasmic dynein and kinesin (Dou et al. 2023). In iPSC-derived human neurons, the introduction of the strongly hyperactive LRRK2-p.R1441H mutation causes significant impairments in autophagosome transport. Knockout of the protein phosphatase 1 H (PPM1H), which opposes LRRK2 activity, produces similar transport defects. However, overexpression of ADP-ribosylation factor 6 (ARF6), a GTPase that regulates the activation of dynein and kinesin, mitigates the transport abnormalities observed in both LRRK2-p.R1441H mutant and PPM1H knockout neurons. These findings suggest that an imbalance between hyperphosphorylated Rabs and ARF6 disrupts the proper transport of autophagosomes, potentially contributing to the development of PD. Third study demonstrated that inhibition of endosomal maturation leads to rapid formation of GTPase-inactivating mutants LRRK2 + endosomes, where LRRK2 phosphorylates its substrate Rabs (Rinaldi et al. 2023). These LRRK2 + endosomes are sustained through positive feedback, reinforcing the membrane localization of both LRRK2 and phosphorylated Rab substrates. Strikingly, cells expressing GTPase-inactivating mutants exhibit a greater number of LRRK2 + endosomes compared to cells expressing kinase-activating mutants, resulting in higher overall levels of phosphorylated Rabs in the cells.

VPS35 and VPS13C are proteins involved in intracellular trafficking and endosomal function. Mutations in the *VPS35* gene were identified as the cause of an autosomal dominant form of PD (Vilariño-Güell et al. 2011; Zimprich et al. 2011) and mutations in the *VPS13C* gene were identified as the cause of autosomal recessive form of PD (Lesage et al. 2016). Sen et al. found that VPS35 is involved in the maturation of early endosomes to late autophagy vesicles, where degradation of mitochondrial DNA (mtDNA) takes place (Sen et al. 2022). The authors found that specific mtDNA damage triggers membrane remodeling and endosomal recruitment in close proximity to mitochondrial nucleoid sub-compartments. The targeting of mitochondrial nucleoids is regulated by the ATAD3-SAMM50 axis. SAMM50, acting as a gatekeeper, influences BAK clustering, controls nucleoid release, and facilitates their transfer to endosomes. Through the action of VPS35, early endosomes mature into late autophagy vesicles, enabling the degradation of mtDNA. This process plays a crucial role in the selective removal of mutation-bearing mtDNA and helps ameliorate mitochondrial dysfunction associated with mtDNA-related diseases (Matsui et al. 2021). Hancock-Cerutti et al. investigated the consequences of VPS13C depletion in HeLa cells (Hancock-Cerutti et al. 2022). The authors found that depletion of VPS13C led to an accumulation of lysosomes with an altered lipid profile. Additionally, they observed activation of the DNA-sensing cGAS-STING pathway, which has recently been implicated in PD pathogenesis, in these cells (Sliter et al. 2018). The activation of the cGAS-STING pathway resulted from two factors: elevated levels of mtDNA in the cytosol and a defect in the degradation of activated STING, which is a lysosome-dependent process. Another group used AlphaFold predictions to complement existing structural information and generated a full-length model of human VPS13C (Cai et al. 2022).

It is well-established that mitochondria, lysosomes, and vesicular transport play crucial roles in the pathogenesis of PD. However, the position occupied by α-synuclein, which is considered a key molecule, within these processes remains unclear, contributing to the ongoing mystery. The following sections will delve into a discussion centered around α-synuclein.

## α-synuclein aggregation, propagation and toxicity in PD pathogenesis

*SNCA* is not only the causative gene for familial PD, but also a risk gene for sporadic PD (Polymeropoulos et al. 1997; Satake et al. 2009; Simón-Sánchez et al. 2009). Protein aggregates seen in PD are mainly composed of α-synuclein and the accumulation and aggregation of α-synuclein are thought to play a central role in the pathogenesis of PD. In the context of the newly identified PD risk gene *GPNMB*, reported in 2022, it has been demonstrated that mutations in this gene lead to an increase in the quantity of α-synuclein within neurons (Weterman et al. 1995; Diaz-Ortiz et al. 2022). In studies of α-synuclein in PD, the most common focus is on its aggregation and toxicity.

Multiple system atrophy (MSA) is another synucleinopathy characterized by α-synuclein inclusions in brain cells. Yang et al. found that the cryo-electron microscopy (cryo-EM) structures of α-synuclein filaments in PD, PD dementia, and DLB consist of a single protofilament called the Lewy fold, which is different from the protofilaments observed in MSA (Yang et al. 2022a). These findings suggest the existence of distinct molecular conformations of α-synuclein in different neurodegenerative diseases. Another study focused on understanding the role of the P1 region (residues 36–42) in the amyloid formation of α-synuclein (Ulamec et al. 2022). Through mutational studies, the authors found that specific mutations in the P1 region, namely Y39A and S42A, prolonged the lag-phase of α-synuclein amyloid formation in vitro and protected against amyloid-induced cytotoxicity in *Caenorhabditis elegans*. They also observed that the L38I mutation accelerated the formation of amyloid fibrils, while L38A had no effect. Interestingly, L38M prevented the formation of amyloid fibrils in vitro and provided protection against proteotoxicity. They demonstrated that the P1 region synergizes with residues in the other regions of α-synuclein to initiate aggregation.

If α-synuclein aggregates are indeed toxic, the idea of intervening in aggregate formation to help treat PD is natural. Kam et al. utilized a mouse model of PD, specifically the α-synuclein preformed fibril (PFF) model, to assess the impact of irisin, an exercise-induced polypeptide, on α-synuclein-induced neurodegeneration (Kam et al. 2022). The results showed that intravenous delivery of irisin via viral vectors reduced the formation of pathological α-synuclein and prevented the loss of dopamine neurons. Irisin treatment also improved motor deficits observed in the mice. In experiments with primary cortical neurons, sustained irisin treatment attenuated α-synuclein-induced toxicity and reduced neuronal cell death. The researchers further demonstrated that irisin enhanced the endolysosomal degradation of pathological α-synuclein, thereby reducing its accumulation. In another study, researchers developed synthetic nanobody libraries in yeast to target pathogenic α-synuclein PFF (Butler et al. 2022). They identified a specific nanobody, PFFNB2, that can recognize α-synuclein PFF but not α-synuclein monomers. PFFNB2 was able to dissociate α-synuclein fibrils. They further demonstrated that using an adeno-associated virus (AAV) encoding EGFP fused to PFFNB2 (AAV-EGFP-PFFNB2), they could inhibit PFF-induced phosphorylation of α-synuclein at serine 129 in mouse primary cortical neurons. In a transgenic mouse model expressing human wild type α-synuclein, intrastriatal injection of PFF led to the propagation of α-synuclein pathology to the cortex, but this propagation was prevented by AAV-EGFP-PFFNB2. These findings suggest that PFFNB2 holds promise for understanding the mechanisms and developing therapies for PD.

α-synuclein aggregates also seem to elicit significant responses in glia. Long et al. utilized structure-based interaction predictions and identified the receptor for advanced glycation end products (RAGE) as a receptor for α-synuclein fibrils on microglia (Long et al. 2022). They confirmed the binding between the V domain of RAGE and the acidic C-terminal residues of α-synuclein using nuclear magnetic resonance (NMR) spectroscopy and mutagenesis techniques. Furthermore, they demonstrated that the binding of α-synuclein fibrils to RAGE triggers neuroinflammation, which can be blocked by depleting RAGE genetically or using the inhibitor FPS-ZM1. Another group found that astrocytes derived from the ventral midbrain (VM) demonstrated the ability to inhibit α-synuclein aggregation and transmission in various in vitro and i*n vivo* models of α-synucleinopathy (Yang et al. 2022b). The therapeutic effect was mediated through the regulation of neuronal α-synuclein proteostasis. VM astrocytes corrected intraneuronal oxidative and mitochondrial stresses, as well as extracellular inflammatory environments, which accelerated pathologic misfolding of α-synuclein. The paracrine factors released from astrocytes promoted disassembly of extracellular α-synuclein aggregates and enhanced neuronal autophagic clearance of α-synuclein. Transplantation of VM astrocytes into the midbrain of PD model mice reduced α-synuclein pathology and protected midbrain dopamine neurons from degeneration.

The above-mentioned reports represent the current topics on α-synuclein aggregation, but recently it is thought that smaller aggregates or oligomers may be more toxic than large aggregates. Morten et al. used an aggregate-activated fluorophore called Amytracker 630 to visualize α-synuclein aggregates (Morten et al. 2022). They found a linear relationship between the size of α-synuclein aggregates and cellular toxicity, with aggregates smaller than 450 ± 60 nm readily penetrating the plasma membrane. They also observed that cell-penetrating aggregates were surrounded by proteasomes, which formed foci to gradually process the aggregates. Another group showed that small aggregates (less than 200 nm) of α-synuclein induced inflammation and permeabilization of single-liposome membranes, while larger aggregates were less toxic (Emin et al. 2022). Analysis of soluble aggregates extracted from post-mortem human brains revealed that the aggregates in PD brains were smaller (less than 100 nm) and more inflammatory compared to aggregates in control brains. Using single-molecule Förster resonance energy transfer (FRET) biosensor, Choi et al. observed the conversion of α-synuclein from a monomeric state to two distinct oligomeric states in neurons, depending on its concentration and sequence (Choi et al. 2022). Three-dimensional FRET-correlative light and electron microscopy (FRET-CLEM) revealed that intracellular seeding events preferentially occur on membrane surfaces, particularly on mitochondrial membranes. The presence of the mitochondrial lipid cardiolipin triggered rapid oligomerization of A53T α-synuclein, and cardiolipin was sequestered within aggregating lipid-protein complexes. These mitochondrial aggregates impaired the activity of complex I and increased the generation of reactive oxygen species (ROS), leading to accelerated oligomerization of A53T α-synuclein, permeabilization of mitochondrial membranes, and ultimately, cell death. Using human iPSCs to generate midbrain dopaminergic neurons, Virdi et al. aimed to identify the early events leading to PD pathology (Virdi et al. 2022). Through single-cell RNA sequencing, proteomics, and functional characterization, they confirmed the molecular and functional identity of the generated neurons. They observed the formation of small β-sheet-rich oligomeric aggregates, preceding the maturation of dopaminergic neurons, in cultures with *SNCA* mutations. Early impairments in intracellular calcium signaling, increased basal calcium levels, and mitochondrial calcium handling were also observed. The study suggests that the formation of intraneuronal oligomers are early critical events in PD pathogenesis.

α-synuclein has been shown to alter its properties through a variety of modifications and interactions. Dopamine metabolite 3,4-dihydroxyphenylacetaldehyde (DOPAL) covalently modifies α-synuclein, leading to its accumulation and impaired clearance in neurons (Masato et al. 2023). This accumulation of DOPAL-modified α-synuclein disrupts neuronal resilience, compromises synaptic integrity, and overwhelms protein quality control pathways in neurites. This progressive disruption of neuronal homeostasis results in the loss of dopaminergic neurons and motor impairment in animal PD models. Jin et al. found through mass spectrometry analysis that tyrosine hydroxylase (TH) converts a specific amino acid (Tyr136) in α-synuclein into dihydroxyphenylalanine (DOPA; Y136DOPA) (Jin et al. 2022). The Y136DOPA modification was observed in dopaminergic neurons of α-synuclein-overexpressing mice and human α-synucleinopathies. The presence of Y136DOPA led to the formation of α-synuclein oligomers, rather than larger fibrillar aggregates, and increased neurotoxicity.

Our research group also recently reported on α-synuclein phosphorylation and its effects. We observed increased T64 phosphorylation in PD models and human PD brains (Matsui et al. 2023). By introducing a phosphomimetic mutation T64D in α-synuclein, we found the formation of distinct oligomers resembling those seen in α-synuclein with the A53T mutation, which is associated with familial PD. This phosphomimetic mutation resulted in mitochondrial dysfunction, lysosomal disorder, cell death in vitro, and neurodegeneration in vivo, suggesting that phosphorylation at T64 contributes to the pathogenesis of PD. Another group investigates the role of phosphorylation in regulating the membrane-binding, vesicle interactions, and aggregation of α-synuclein (Reimer 2022). The researchers demonstrate that Protein kinase R (PKR) can phosphorylate α-synuclein at specific Ser/Thr residues located in the membrane-binding region crucial for vesicle interactions. Phosphorylation, particularly at T64 and T72, reduces α-synuclein’s binding to lipid membranes, affecting its overall attachment to brain vesicles. A phosphomimetic mutant of α-synuclein at T64 and T72 exhibits decreased vesicle affinity, promotes vesicle clustering, inhibits α-synuclein oligomerization, and prevents α-synuclein fibrillation and trans-synaptic spreading of aggregated α-synuclein in cultured hippocampal slices.

These reports suggest that attempts to reduce α-synuclein oligomer may be useful for treatment of PD. Nim et al. aimed to identify protein-protein interaction inhibitors that can reduce the levels of α-synuclein oligomers and their associated cytotoxicity (Nim et al. 2023). Through a high-throughput peptide screen, the researchers discovered that a peptide inhibitor disrupting the interaction between the C-terminal region of α-synuclein and CHMP2B (a component of the ESCRT-III complex) was the most effective. They demonstrated that α-synuclein interferes with endolysosomal activity through this interaction, inhibiting its own degradation. Conversely, the peptide inhibitor restores endolysosomal function, leading to decreased α-synuclein levels in various models, including human cells with disease-causing α-synuclein mutations. Additionally, the peptide inhibitor protected dopaminergic neurons from α-synuclein-induced degeneration in models of PD.

Cell-to-cell propagation of pathological proteins play a role in the progression of neurodegenerative diseases (Guo and Lee. 2014). Helwig et al. investigated the relationship between neuronal activity and the transfer of α-synuclein between neurons (Helwig et al. 2022). They found that hyperactivity in a mouse model enhanced the transfer of α-synuclein, while hypoactivity attenuated it. Hyperactivity was associated with increased oxidative and nitrative reactions, accumulation of nitrated α-synuclein, and increased protein aggregation. They identified mitochondria as potential sources of reactive oxygen and nitrogen species within hyperactive neurons. Another study highlights the positive impact of treadmill exercise in reducing the spreading of α-synuclein in the brain and protecting dopaminergic neurons in a mouse model of PD (Dutta et al. 2022). The researchers found that the administration of α-synuclein PFF led to increased α-synuclein propagation and loss of dopaminergic neurons. However, regular treadmill exercise effectively reduced α-synuclein propagation and protected dopaminergic neurons in these mice. They discovered that treadmill exercise activates the peroxisome proliferator-activated receptor α (PPARα) in the brain, which stimulates the biogenesis of lysosomes through the activation of transcription factor EB (TFEB). In mice lacking PPARα, treadmill exercise was unable to stimulate TFEB or reduce α-synucleinopathy. Additionally, treatment with fenofibrate, a PPARα agonist, decreased α-synucleinopathy. Using similar PFF model, computational model successfully predicted the propagation patterns of α-synuclein from various brain regions and even estimated their origins (Dadgar-Kiani et al. 2022). The PTMs discussed in the previous section may also affect the propagation of α-synuclein. A report in Nature Neuroscience reported that the phosphorylation of soluble, nonpathological α-synuclein at specific sites significantly affects the propagation of pathological α-synuclein (Zhang et al. 2023). The authors identified several new PTMs of soluble α-synuclein, including phosphorylation and acetylation, which influenced the transmission of pathological α-synuclein in a site- and conformation-specific manner. Furthermore, the phosphorylation of soluble α-synuclein could modulate the seeding properties of pathological α-synuclein.

The molecular and cell biological mechanisms by which α-synuclein leaves one cell and enters another have not yet been determined. Herman et al. administered extracellular vesicles from the CSF of PD patients or patients with other neurodegenerative disorders to healthy mice through the nasal route (Herman et al. 2023). After three months, the mice treated with extracellular vesicles from PD displayed symptoms resembling early stages of PD, including impaired sense of smell, motor deficits, and increased anxiety levels. Histochemical analysis of their midbrains revealed widespread α-synuclein aggregations, degeneration of dopaminergic neurons and neuroinflammation. These findings indicate that intranasally administered extracellular vesicles derived from the CSF of PD can propagate α-synuclein aggregation in healthy mice, leading to the development of PD-like symptoms and pathological features. Another group utilized various microscopy techniques and subcellular fractionation to show that α-synuclein PFFs are rapidly internalized and targeted directly to lysosomes within 2 min (Bayati et al. 2022). The uptake of PFFs is disrupted by macropinocytic inhibitors and does not follow the classical endosomal pathways. Immunogold-labeled PFFs are observed at the curved edge of membrane ruffles, in newly formed macropinosomes, multivesicular bodies, and lysosomes. While most fibrils remain in lysosomes, some are transferred to neighboring cells along with exosome markers, suggesting a unique mechanism of cell-to-cell propagation. Xie et al. aimed to investigate the mechanism of release for pathogenic aggregates of α-synuclein in synucleinopathies such as PD (Xie et al. 2022). They generated a new mouse model to isolate neuronal lysosomes and established a culture model where α-synuclein aggregates were produced within neurons. The study demonstrated that pathogenic α-synuclein species accumulate within neuronal lysosomes in mouse brains and primary neurons. Furthermore, the researchers found that neurons release these pathogenic α-synuclein aggregates through a process called SNARE-dependent lysosomal exocytosis. The released aggregates were non-membrane enveloped and capable of seeding. The release of α-synuclein aggregates was dependent on neuronal activity and cytosolic calcium levels. Our group also explored the mechanisms underlying α-synuclein transmission. Following establishment of a mouse model demonstrating the extensive spread of α-synuclein pathology from the olfactory bulb by injection of α-synuclein PFF (Uemura et al. 2021), we showed that inhibition of the Ca2+-calmodulin-calcineurin signaling blocked the neuronal uptake of α-synuclein preformed fibrils via macropinocytosis (Ueda et al. 2023). Thus Ca2+-calmodulin-calcineurin signaling can be a potential therapeutic target for PD. We further reported that perampanel, an AMPA receptor antagonist, inhibited α-synuclein pathology by modulating neuronal activity, offering an innovative approach for disease modification in PD (Ueda et al. 2021). Moreover, we created a marmoset model of Lewy body disease by injection of α-synuclein to the olfactory bulb. Extensive glucose hypometabolism in the occipital cortex in the marmoset model shown by FDG-PET suggests that this primate model recapitulates an important aspect of human PDD/DLB (Sawamura et al. 2022). Together, these studies provide a comprehensive perspective on α-synuclein transmission and its implications for understanding and treating PD, from fundamental mechanisms to therapeutic prospects.

There is substantial experimental and clinical evidence suggesting that the formation of oligomers and aggregates by α-synuclein can be detrimental in certain cases. Moreover, experimental evidence supports the likelihood of α-synuclein propagation, and clinical observations supporting this phenomenon cannot be dismissed. However, it is crucial to note that the definitive occurrence of these events in the patient’s body and brain is yet to be confirmed for both aspects.

## Physiological functions of α-synuclein in PD pathogenesis

We have discussed many topics on the pathological conditions that α-synuclein induces, but in fact the major physiological functions of α-synuclein are still inconclusive. α-synuclein is localized to the axon terminals of neurons. While it is suggested to interact with membranes, when expressed in cultured cells, it generally exhibits a diffuse distribution. In mice, the loss of α-synuclein has minimal effects on behavior and neurodegeneration, but there are indications that it may be involved in processes such as vesicle release at neuronal synapses (Sulzer and Edwards 2019).

A paper in *Nature Neuroscience* reveals that synucleins are necessary for the release of endocannabinoids (Albarran et al. 2023). The researchers demonstrate that endocannabinoid-dependent synaptic plasticity is blocked when synucleins are deleted, and this block can be reversed by introducing wild-type α-synuclein. The findings indicate that endocannabinoids are released postsynaptically through a synuclein-dependent and SNARE-dependent mechanism. The deletion of synucleins specifically impairs endocannabinoid release, as observed through electrophysiological recordings and direct optical monitoring. The study suggests that synucleins, known for their role in regulating vesicle lifecycle, facilitate endocannabinoid release through a membrane interaction mechanism. Another group found that multiple homozygous loss-of-function mutations in *DAGLB* were associated with early onset autosomal recessive PD by combining WES and other analyses (Liu et al. 2022). DAGLB is the main enzyme responsible for producing endocannabinoid 2-arachidonoyl-glycerol (2-AG) in dopaminergic neurons of the substantia nigra (SN) in humans and mice. In mice, the levels of 2-AG in the SN were strongly correlated with motor performance during locomotor skill acquisition. Knockdown of mouse Daglb in nigral dopaminergic neurons reduced 2-AG levels, impaired locomotor skill learning, and particularly affected across-session learning. Conversely, inhibiting the degradation of 2-AG increased its levels, enhanced dopaminergic neuron activity and dopamine release in the SN, and rescued the deficits in locomotor skill learning. These findings demonstrate that deficiency in DAGLB contributes to the development of PD.

On the other hand, there are several reports suggesting that it is a completely different story from synaptic function. A study in Cell investigates the interaction between α-synuclein, and processing bodies (P-bodies), organelles involved in mRNA turnover (Hallacli et al. 2022). The researchers demonstrate that α-synuclein can bind to both cellular membranes and P-bodies, with the N terminus of α-synuclein playing a crucial role in this binding. Abnormal accumulation of α-synuclein disrupts the interaction between α-synuclein and decapping proteins, leading to impaired mRNA decay kinetics in PD-relevant pathways. Modulating P-body components affects α-synuclein toxicity, and human genetic analysis supports the relevance of these interactions in PD. Monogue et al. found that α-synuclein is necessary for the expression of interferon-stimulated genes in neurons during viral infection (Monogue et al. 2022). In human α-synuclein knockout neurons treated with type 1 interferon, the induction of interferon-stimulated genes was impaired. Additionally, α-synuclein accumulated in the nucleus of interferon-treated human neurons, and its expression was required for the phosphorylation of STAT2, a key player in interferon signaling. The researchers observed co-localization of activated STAT2 with α-synuclein following type 1 interferon stimulation in neurons.

It is currently inconclusive which role of α-synuclein is associated with the pathophysiology of PD, whether there are other crucial functions, or if α-synuclein’s physiological functions are unrelated to PD. Due to the extensive research on PD, studies on the functions of α-synuclein often focus on neurons. However, it is intriguing to note that α-synuclein is highly expressed not only in neurons but also in cells of the hematopoietic system and certain malignant tumors (Araki et al. 2018; Zanotti et al. 2023). At this stage, definitive conclusions cannot be drawn. Understanding the physiological function of α-synuclein will be necessary for a full understanding of PD. Further progress in elucidating both the pathophysiology and physiological function of α-synuclein is expected.

## Conclusion

In conclusion, significant progress has been made in elucidating the etiology of PD: the genetics and epigenetics of PD have been extensively studied, and several novel genetic variants associated with PD risk and pathogenesis have been identified. Molecular and cytopathological studies have revealed the role of protein aggregation, neurodegeneration and mitochondrial-lysosomal dysfunctions in the pathogenesis of PD. Despite these advances, however, much work remains to be done, including understanding of underlying mechanisms, and development of more effective therapies that can slow or halt disease progression. Nevertheless, since progress has been made over this 12-month period beyond the content of this review, we may expect some great discoveries in the near future. Remarkable advances in analytical technology support PD research, and we look forward to the breakthroughs that will result from these advances.

## Data Availability

This is a review article based on published literature, no original data was produced, no experiments were made.
